# Differential Expression of hERG1 Channel Isoforms Reproduces Properties of Native *I*_Kr_ and Modulates Cardiac Action Potential Characteristics

**DOI:** 10.1371/journal.pone.0009021

**Published:** 2010-02-02

**Authors:** Anders Peter Larsen, Søren-Peter Olesen

**Affiliations:** The Danish National Research Foundation Centre for Cardiac Arrhythmia, Department of Biomedical Sciences, University of Copenhagen, Copenhagen, Denmark; University of Cincinnati, United States of America

## Abstract

**Background:**

The repolarizing cardiac rapid delayed rectifier current, *I*_Kr_, is composed of ERG1 channels. It has been suggested that two isoforms of the ERG1 protein, ERG1a and ERG1b, both contribute to *I*_Kr_. Marked heterogeneity in the kinetic properties of native *I*_Kr_ has been described. We hypothesized that the heterogeneity of native *I*_Kr_ can be reproduced by differential expression of ERG1a and ERG1b isoforms. Furthermore, the functional consequences of differential expression of ERG1 isoforms were explored as a potential mechanism underlying native heterogeneity of action potential duration (APD) and restitution.

**Methodology/Principal Findings:**

The results show that the heterogeneity of native *I*_Kr_ can be reproduced in heterologous expression systems by differential expression of ERG1a and ERG1b isoforms. Characterization of the macroscopic kinetics of ERG1 currents demonstrated that these were dependent on the relative abundance of ERG1a and ERG1b. Furthermore, we used a computational model of the ventricular cardiomyocyte to show that both APD and the slope of the restitution curve may be modulated by varying the relative abundance of ERG1a and ERG1b. As the relative abundance of ERG1b was increased, APD was gradually shortened and the slope of the restitution curve was decreased.

**Conclusions/Significance:**

Our results show that differential expression of ERG1 isoforms may explain regional heterogeneity of *I*_Kr_ kinetics. The data demonstrate that subunit dependent changes in channel kinetics are important for the functional properties of ERG1 currents and hence *I*_Kr_. Importantly, our results suggest that regional differences in the relative abundance of ERG1 isoforms may represent a potential mechanism underlying the heterogeneity of both APD and APD restitution observed in mammalian hearts.

## Introduction

The molecular correlate of the cardiac rapid delayed rectifier current, *I*_Kr_, is the ERG1 (Kv11.1) ion channel [1], [2]. Mutations in the human variant of ERG1 (hERG1) has been linked to the long QT [3] and short QT syndromes [4]. Two isoforms of ERG1, ERG1a and ERG1b, have been detected on the protein level in both rat, canine and human cardiac tissue [5]. The isoforms differ only in the N-terminal region. Compared to ERG1a, the N-terminus of ERG1b is 340 amino acids shorter. However, the initial 36 residues of ERG1b are unique to this isoform [6], [7]. Quantification of mRNA from healthy human hearts have shown that hERG1b accounts for 10–20% of the total hERG1 transcripts [8]. Very recently, a mutation in the unique N-terminus of hERG1b was discovered in a patient with long QT syndrome [9] highlighting the importance of this isoform in cardiac repolarization. However, little is known about regional differences in the relative abundance of hERG1a and hERG1b or the potential functional consequences thereof.

Gintant [10] used voltage ramps to characterize *I*_Kr_ transients in isolated canine cardiomyocytes. The *I*_Kr_ transient showed only little variation in the peak current density but a large variation (20–30 mV) in the membrane potential at which the current peaked. This variation in ‘peak potential’ was evident in both atrial, ventricular and purkinje fiber cells. These observations suggest that the kinetic properties of *I*_Kr_ are different between individual cardiac cells. Theoretically, in terms of ERG1 channel gating, differences in kinetics of recovery from inactivation and kinetics of deactivation could explain such a variation in ‘peak potential’. Interestingly, it has been shown that the deactivation rate of *I*_Kr_ is faster in epicardial than in midmyocardial cells obtained from canine left ventricular muscle [11] supporting that regional differences in the kinetics of *I*_Kr_ exist in mammalian hearts.

In heterologous expression systems, heteromeric ERG1a/b, homomeric ERG1a and homomeric ERG1b channels differ in their kinetic properties [6], [8], [9], [12]. Potentially, by varying the relative abundance of ERG1 isoforms, the macroscopic kinetics of the resulting ERG1 current can be modified. We therefore hypothesized that the variation in ‘peak potential’ of the *I*_Kr_ transient observed in native cardiac cells [10] can be explained by differences in macroscopic channel kinetics caused by a variation in the relative abundance of ERG1 isoforms. As the functional consequences of regional differences in the relative abundance of ERG1 isoforms are largely unknown we explored this as a potential mechanism underlying native heterogeneity of action potential characteristics.

We find that the characteristics of native *I*_Kr_ transients can be reproduced in heterologous expression systems by differential expression of hERG1a and hERG1b isoforms. Furthermore, we describe how differential expression of hERG1a and hERG1b may in turn modulate both action potential duration (APD) and restitution properties.

## Methods

### DNA Constructs

cDNAs encoding hERG1a (acc. number NM_000238) and hERG1b (acc. number NM_172057) were inserted into either pcDNA3 (for mammalian expression) or into pXOOM [13] (for expression in *X. laevis* oocytes) plasmids. The hERG1b clone was obtained from A. Arcangeli (Università degli Studi di Firenze, Italy).

### Expression in HEK293 Cells

HEK293 cells were maintained in DMEM (Invitrogen, Carlsbad, CA, USA) supplemented with 10% fetal bovine serum and 5% CO2 at 37°C. At 50–60% confluency, cells were transiently transfected with a total of 2 µg of cDNA using Lipofectamine (Invitrogen, Carlsbad, CA, USA) according to the manufacturer's instructions. For co-transfection, 1 µg of each cDNA was used. 0.3 µg of eGFP in pcDNA3 was co-transfected to identify successfully transfected cells. 48 hours after transfection, the cells were trypsinized and transferred to cover slips for experiments.

### Expression in *X. laevis* Oocytes

cRNA for injection was prepared from the linearized DNA constructs using the T7 m-Message Machine kit (Ambion, Austin, TX, USA) according to the manufacturer's instructions. RNA concentrations were quantified using a Nanodrop ND-1000 spectrophotometer (NanoDrop Technologies, Wilmington, DE, USA) and RNA quality was checked by gel electrophoresis. *X. laevis* oocytes were either purchased from Ecocyte Bioscience (Castrop-Rauxel, Germany) or prepared in-house. In the latter case *X. laevis* surgery and oocyte treatment were performed according to the guidelines of the Danish National Committee for Animal Studies. For co-expression of hERG1a and hERG1b cRNAs were mixed in different molar ratios (20%, 40%, 60% and 80% hERG1b) before injection. After injection the oocytes were kept in Kulori solution (in mM: 87 NaCl, 4 KCl, 1 MgCl_2_, 1 CaCl_2_, 5 HEPES, pH 7.4) at 19°C for 2–3 days before experiments were performed.

### Electrophysiological Procedures

Measurements on HEK293 cells were performed in the whole-cell patch clamp configuration using an EPC-9 patch clamp amplifier (HEKA Elektronik, Lambrecht/Pfalz, Germany). The glass pipettes for the recording electrodes had a tip resistance of 1.5–2.5 MOhm when filled with intracellular solution. The series resistance recorded in the whole-cell configuration was 2–8 MOhm and was compensated (80%). The extracellular solution contained (in mM): NaCl 140, KCl 4, CaCl_2_ 2, MgCl_2_ 1 and HEPES 10, pH 7.4. The intracellular solution used in the pipettes contained (in mM): KCl 110, EGTA 10, CaCl_2_ 5.17, MgCl_2_ 1.42, K_2_ATP 4, HEPES 10, pH 7.2.

Measurements on *X. laevis* oocytes were performed with the two-electrode voltage-clamp technique using a Dagan CA-1B amplifier (Dagan Corporation, Minneapolis, MN, USA). The recordings were performed under continuous superfusion with Kulori solution. The glass pipettes for the recording electrodes were filled with 2 M KCl and had tip resistances of 0.5–2.5 MOhm.

All recordings were performed at room temperature and data was acquired using the Pulse software (HEKA Elektronik, Lambrecht/Pfalz, Germany). The time constant of activation at 0 mV was determined using the standard envelope of tails protocol, measuring the peak tail current at either −60 mV or −100 mV. A single-exponential function was fitted to the normalized peak tail currents to obtain τ_act_. The time constants of channel deactivation (τ_slow_ and τ_fast_) were obtained by fitting a double-exponential function to tail current traces measured at −60 mV following a voltage step to +20 mV for 1000 ms to achieve maximal activation. Similarly, the time constant of recovery from inactivation (τ_rec_) at −60 mV was determined from the initial rising phase of the tail currents recorded with this protocol. The extrapolated fit of the deactivation process was subtracted from the initial rising phase of the tail currents. The difference between the extrapolated values and the recorded current values represents the time course of recovery from inactivation. The time constant of this process was estimated by fitting a single-exponential function to the resulting curve. Please see the Results section and figure legends for details on the voltage ramp protocols. For all protocols, the holding potential was −80 mV.

### Computational Modeling

All simulations were performed using the COR program [14]. The built-in CVODE integrator was used with a time step of 0.01 ms. All models were encoded in CellML 1.0 (http://www.cellml.org/).

**Figure 1:**
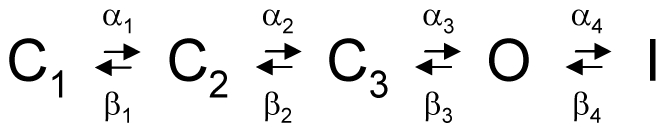
Topology of the hERG1 Markov model. The hERG1 Markov model developed by Fink et al. [15], consists of three closed states (C_1_-C_3_), an open state (O) and an inactivated state (I). Allowed transitions between states are indicated with arrows. The transition rates are indicated above or below the corresponding transitions.

We adopted the Markov model formulation of hERG1 as described by Fink et al. [15]. The model includes three closed states (C_1_-C_3_), an open state (O) and an inactivated state (I). The topology of the model is shown in figure 1. The model has been shown to reproduce data recorded both at room temperature and at physiological temperature reasonably well. The hERG1 Markov simulations were performed at 296 K with 4 mM extracellular potassium. In our simulations we modified the transition rate of activation α_3_ (transition from the closed state C_3_ to the open state O), the transition rate of deactivation β_3_ (transition from the open state O to the closed state C_3_) and the transition rate of recovery from inactivation β_4_ (transition from the inactivated state I to the open state O).

For single cell action potential (AP) simulations we used the ten Tusscher model (TT) of the human ventricular epicardial cell [16]. The model was obtained from the CellML repository (http://www.cellml.org/models/tentusscher_panfilov_2006_version04) on the 11^th^ of April 2009 (Note: after the model was downloaded the CellML website has been restructured and the model can now be found at http://models.cellml.org/exposure/de5058f16f829f91a1e4e5990a10ed71). This version of the model has reached curation level 2 indicating that it qualitatively reproduces the results published in the original paper (see http://www.cellml.org/repository-info/info for details on the curation procedure). We exchanged the original Hodgkin-Huxley formulation of *I*_Kr_ for the hERG1 Markov model described above [15]. In the modified model (TTF) we adjusted *G*_Kr,max_ by a factor of 0.3 to 0.046 nS/pF. Otherwise, no changes were made to the downloaded model. To ensure steady-state conditions the ventricular cell model was paced at a basic cycle length (BCL) of 1000 ms for 500 beats prior to any modification of hERG1 kinetics. After the kinetic modifications the models were paced for another 100 beats (BCL 1000 ms) to ensure that a new steady-state was reached. APD restitution was determined using the standard S1-S2 protocol. After reaching steady-state (BCL 1000 ms) a premature stimulus (S2) was introduced at progressively shorter diastolic intervals (DI). The APD restitution curve was obtained by plotting APD against the preceding DI. A single-exponential function was fitted to each restitution curve and the slope was calculated by differentiation of the fitted curve.

### Data Analysis

Data analysis was performed with Igor Pro (Wavemetrics, Lake Oswego, OR, USA), GraphPad Prism (GraphPad Software Inc., San Diego, CA, USA) and R (http://www.R-project.org) software. Statistical comparisons were performed using ANOVA followed by Tukey's procedure or an appropriate set of custom-defined contrasts. A P-value of less than 0.05 was considered statistically significant. Unless otherwise stated, data are represented as mean ± SEM.

The electrophysiological experiments were not designed to compare the absolute current levels between hERG1a and hERG1b channels. In order to compare data independently of differences in transfection efficiency and/or translation/trafficking processes, we normalized the current levels to a value (N) proportional to the number of expressed channels as described by Sale et al. [9]. Briefly, we subjected cells/oocytes to a depolarizing voltage step to +40/+20 mV for 1000 ms to achieve maximal activation of hERG1 channels followed by a step to −60 mV for 3 s to record channel deactivation. A double-exponential function was fitted to the current decay at −60 mV. By extrapolation back to the beginning of the −60 mV voltage step we obtained an estimate of N. This normalization is valid under the assumption that the single channel conductances are identical. It has been shown, that for hERG1a single channel conductance does not depend on the length of the N-terminus [17]. As the only difference between hERG1a and hERG1b is the N-termini the assumption seems justified.

## Results

### Differential Expression of hERG1a and hERG1b Modulates the ‘Peak Potential’

**Figure 2:**
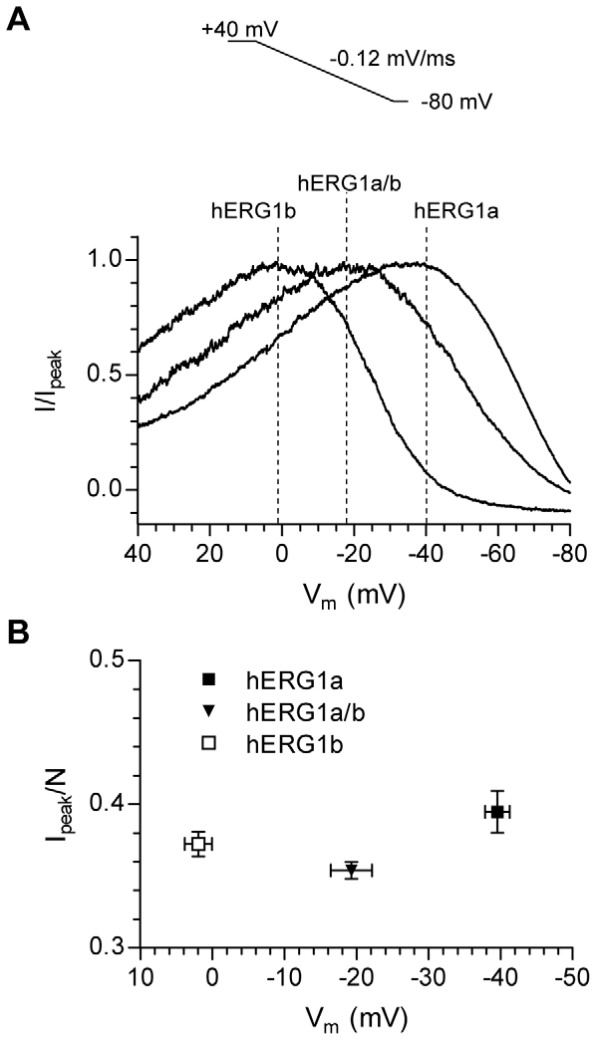
The relative abundance of hERG1a and hERG1b determines the ‘peak potential’ in mammalian cells. A, Representative recordings of hERG1a, hERG1b and hERG1a/b channels expressed in HEK293 cells are shown. Currents were recorded using the protocol depicted in the inset. Only currents recorded during the ramp are shown. For comparison, currents were normalized to the maximum peak current during the ramp and plotted against the command voltage. The dotted lines indicate the potential where the currents peaked (‘peak potential’) and are also used to indicate the expressed channels. B, Summary data of the variation in ‘peak potential’. For comparison, the peak amplitude was normalized to an estimate of the number of channels (N) and plotted as a function of the ‘peak potential’.

In order to address whether differential expression of hERG1a and hERG1b was able to modulate the ‘peak potential’, i.e. the potential where the current peaks during a repolarizing voltage ramp, we transfected HEK293 cells with either hERG1a, hERG1b or both channel subunits. The cells were clamped at +40 mV for 200 ms to activate the channels and then repolarized to −80 mV using a ramp with a slope of −0.12 mV/ms. Representative currents recorded during the ramp are shown in figure 2A. Currents were normalized to the maximum peak current for comparison. The data show that expression of hERG1b was able to shift the ‘peak potential’ significantly towards more depolarized potentials. Summary data are shown in figure 2B. To compare peak amplitudes independently of expression levels data was normalized to a value, N, proportional to the number of expressed channels (see Methods for details). For hERG1a channels the ‘peak potential’ was −39.6±1.7 mV, for hERG1a/b channels it was shifted to −19.3±2.9 mV and for hERG1b channels it was 1.9±1.9 mV (P<0.05 for all comparisons). The peak amplitudes, normalized to N, were 0.39±0.01 (hERG1a), 0.35±0.01 (hERG1a/b, P<0.05 compared to hERG1a) and 0.37±0.01 (hERG1b).

**Figure 3:**
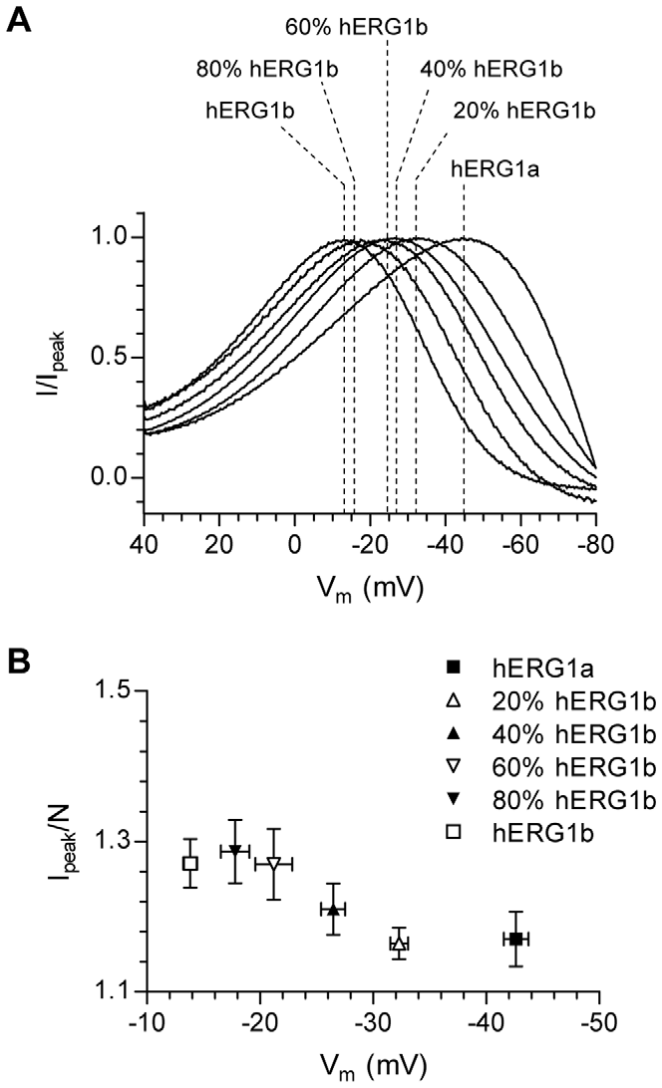
The ‘peak potential’ is directly dependent on the relative abundance of hERG1 isoforms. A, Representative recordings from *X. laevis* oocytes injected with different ratios of hERG1 isoforms. The same protocol as shown in figure 2A was used. Only currents recorded during the ramp are shown. For comparison, currents were normalized to the maximum peak current during the ramp and plotted against the command voltage. The dotted lines indicate the potential where the currents peaked (‘peak potential’) and are also used to indicate the isoform ratio (in % of hERG1b abundance). B, Summary data of the variation in ‘peak potential’. For comparison, the peak amplitude was normalized to N and plotted as a function of the ‘peak potential’.

To verify the relationship between ‘peak potential’ and relative abundance of hERG1b we repeated the experiment in *X. laevis* oocytes. In the oocyte expression system, cRNA is injected directly into the oocytes and hence, the relative amounts of cRNA can be controlled very precisely. In figure 3A currents recorded from oocytes injected with different ratios of hERG1a and hERG1b (indicated as % of hERG1b abundance) are shown. The voltage protocol and normalization of data are identical to what is described above for figure 2A. The data show that as the relative abundance of hERG1b was increased, the ‘peak potential’ was gradually shifted towards more depolarized potentials, demonstrating that there was indeed a very good correlation between the ‘peak potential’ and the relative abundance of hERG1b. The results of the oocyte experiments are summarized in figure 3B. The ‘peak potentials’ were −42.6±1.1 mV (hERG1a; 0% hERG1b), −32.3±0.8 mV (20% hERG1b), −25.4±1.1 mV (40% hERG1b), −21.2±1.6 mV (60% hERG1b), −17.8±1.3 (80% hERG1b) and −13.8±0.6 mV (100% hERG1b) (P<0.05 for all comparisons except for 60% vs. 80% hERG1b and 80% vs. 100% hERG1b). When normalized to N there was no significant variation in the peak amplitude. The values were 1.17±0.03 (hERG1a; 0% hERG1b), 1.16±0.02 (20% hERG1b), 1.21±0.03 (40% hERG1b), 1.27±0.05 (60% hERG1b), 1.29±0.04 (80% hERG1b) and 1.27±0.03 (100% hERG1b).

### Macroscopic Kinetics of hERG1 Channels Are Dependent on the Relative Abundance of hERG1a and hERG1b

Previously, it has been shown that hERG1a and hERG1b channels vary in kinetics of activation, deactivation and recovery from inactivation, i.e. the rates are increased by hERG1b, whereas no change in inactivation kinetics are observed.[8], [9] To further verify our hypothesis we determined the kinetics of activation, deactivation and recovery from inactivation for hERG1 channels expressed in *X. laevis* oocytes using the same ratios of hERG1a to hERG1b as described above. In table S1 the time constants of these processes are given. Based on the average values, the relative changes in the time constants were calculated (table S1). Interestingly, linear correlations could be obtained for the relative changes in both τ_fast_ (r^2^ = 0.95), τ_slow_ (r^2^ = 0.99) and τ_rec_ (r^2^ = 0.94) as a function of injected hERG1b cRNA (figure S1A-C). It was not possible to obtain a reliable estimate of τ_rec_ for hERG1b channels alone due to a significant overlap with the transient capacitative current. Instead, the relative change in τ_rec_ for hERG1b compared to hERG1a was calculated by extrapolation from the linear fit over the range 0–80% hERG1b (figure S1C). In contrast to τ_fast_ and τ_rec_ the relative change in the time constant of activation was not linear. Rather, with the exception of 40% hERG1b the time constant was increased by approximately 1.5 fold for all ratios compared to hERG1a alone (figure S1D). For 40% hERG1b the time constant of activation was increased almost 2-fold. However, statistical analysis showed that although significantly different from hERG1a (0% hERG1b, P<0.05 for all comparisons) the time constants of activation for 20–100% hERG1b were not significantly different from each other.

The possibility exists, that the intermediate kinetics observed in co-injected oocytes could arise from a channel population consisting of a mixture of distinct homomeric hERG1a and hERG1b channels. To address this issue, the observed tail currents recorded using the deactivation protocol from oocytes injected with 20–80% hERG1b were compared with the expected theoretical current decay assuming the formation of only homomeric channels. Averages of the observed current decay for 20–80% hERG1b were calculated using the corresponding fitted time constants and their relative weights (table S1). The theoretical current decay was calculated as a weighted algebraic summation of the deactivation kinetics of hERG1a and hERG1b homomeric channels. Figure S2 shows the normalized decay of both the observed and theoretical situation at the four different isoform ratios. For all four comparisons, the (averaged) observed currents decay faster than the theoretical curves. Thus, the observed currents can not be explained by the presence of homomeric channels only. Similarly, the finding that the relative increase in activation kinetics did not change gradually as the relative abundance of hERG1b cRNA was increased also argues that the channel population upon co-expression of hERG1a and hERG1b consists of heteromeric channels rather than two separate pools of homomeric hERG1a and hERG1b channels.

### A hERG1 Markov Model Reproduces the Experimental Data

**Figure 4:**
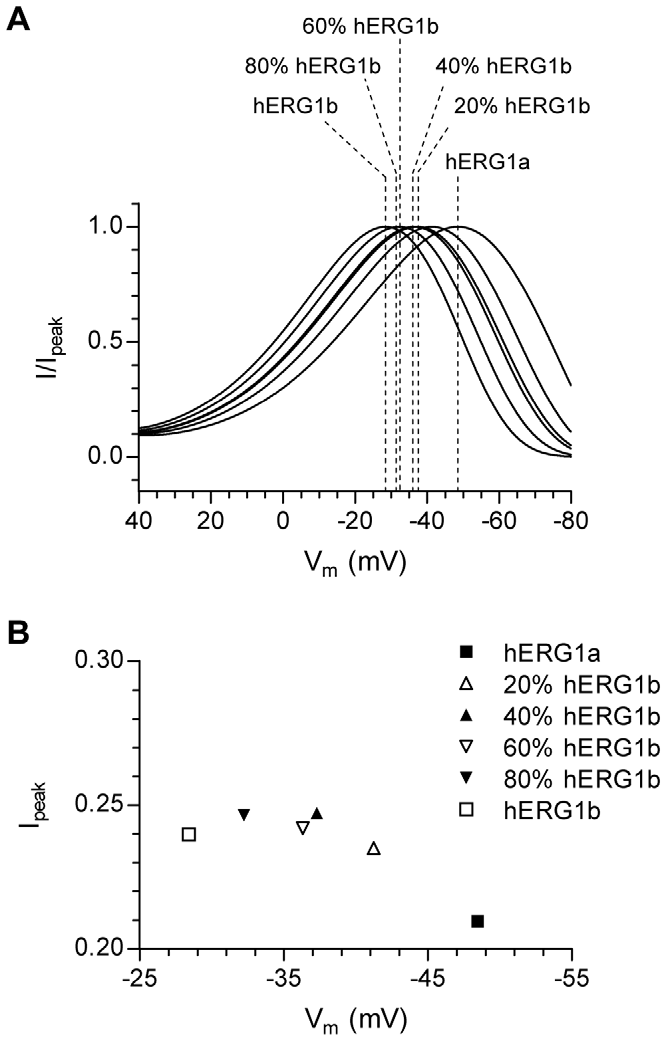
The experimental data is reproduced by a hERG1 Markov model. A, The effect of changes in transition rates, representing a gradual increase in the relative abundance of hERG1b, on simulated hERG1 currents. The transition rates of hERG1 channel activation (α_3_), deactivation (β_3_) and recovery from inactivation (β_4_) were modified to represent a gradual increase in the relative abundance of hERG1b (see table S2 for details). The model was subjected to the same voltage protocol as in figure 2A. Only currents recorded during the ramp are shown. For comparison, currents were normalized to the maximum peak current during the ramp and plotted against the command voltage. The dotted lines indicate the potential where the currents peaked (‘peak potential’) and are also used to indicate the isoform ratio (in % of hERG1b abundance). B, The variation in ‘peak potential’ amplitude is shown as a function of ‘peak potential’. The data were not normalized as the number of channels in the model remained constant.

The Markov model of hERG1, developed by Fink et al. [15], is able to reproduce hERG1 currents recorded both at room temperature as well as at physiological temperature. The relative changes in kinetics determined from the oocyte experiments reflecting different ratios of hERG1a and hERG1b were incorporated into the Markov model to investigate whether the model could reproduce the experimental findings. The factors by which the rate constants were changed are given in table S2. Like most hERG1 Markov models, the present model uses a single-exponential function to describe the transition from the open to the first closed state (O to C, transition rate β_3_) while the deactivation process experimentally is most commonly best fit with a double-exponential function. Changes in the model deactivation kinetics were based on the relative changes in the fast component of deactivation obtained from the experiments. The model was subjected to the same voltage clamp protocol that was used in the *in vitro* experiments (see inset in Figure 2A). The resulting simulated currents are shown in figure 4A. Increasing the rate constants of activation, deactivation and recovery from inactivation by the same factors as obtained from the oocyte experiments gradually shifted the ‘peak potential’ from −48.4 mV (representing hERG1a) to −28.4 mV (representing hERG1b). The overall shape of the currents were very similar to the recordings obtained for hERG1 channels expressed in both mammalian cells and oocytes (compare figure 4A to figures 2A and 3A). The peak amplitude is plotted against the ‘peak potential’ in figure 4B. The changes in peak amplitude followed a pattern similar to the one observed in the oocyte experiments (compare to figure 3B).

### Action Potential Characteristics Are Modulated by the Relative Abundance of hERG1a and hERG1b

To examine the potential functional effects of changes in the relative abundance of hERG1a and hERG1b on the cellular level we substituted the original Hodgkin-Huxley formulation of *I*_Kr_ in the TT model of the human ventricular cardiomyocytes [16] for the Fink Markov model of the hERG1 current. We refer to the modified model as TTF. After adjustment of the maximum conductance of *I*_Kr_ (*G*_Kr,max_) there were only minor differences in the shape of *I*_Kr_ when comparing the two models (data not shown). Steady-state APD_90_ (BCL 1000ms) was 303 ms and 302 ms for the TT and the modified TTF models respectively.

**Figure 5:**
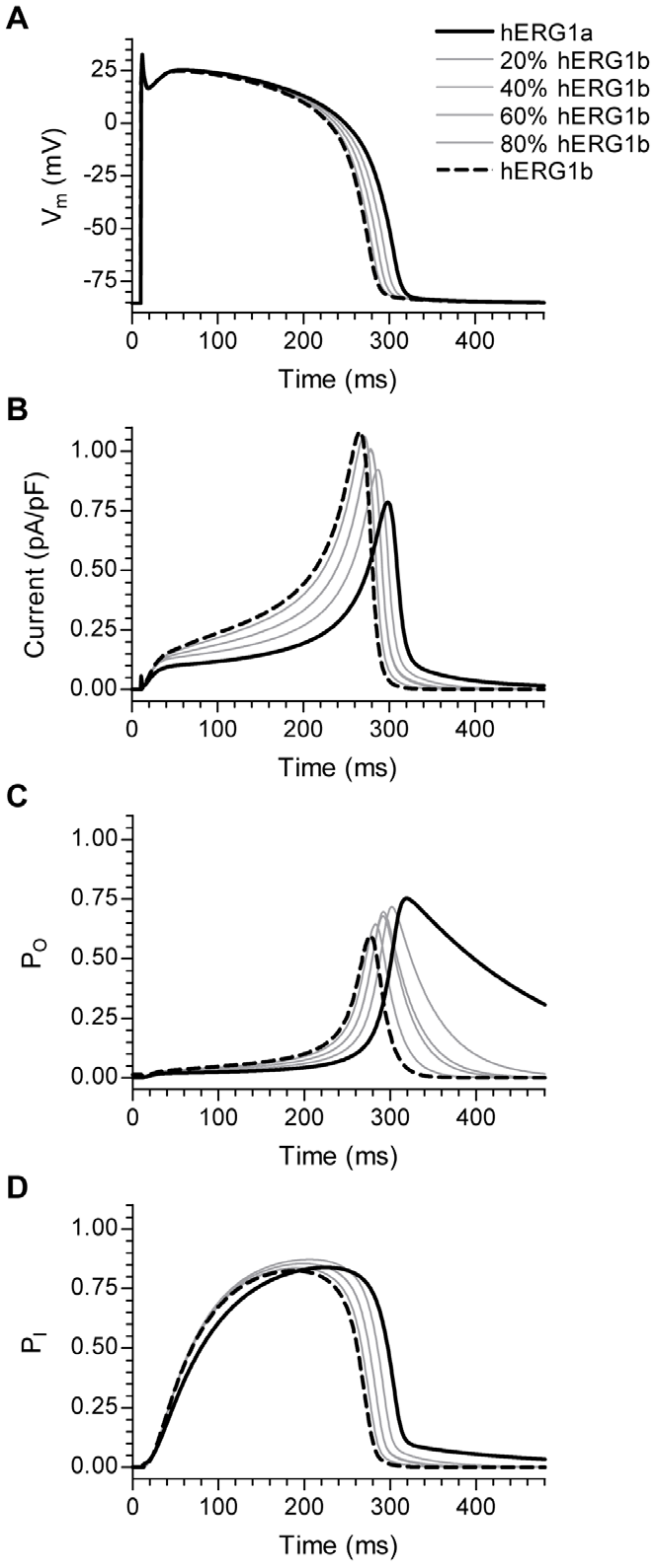
Action potential duration is modulated by the relative abundance of hERG1a and hERG1b. A, Steady-state action potentials simulated by the modified ten Tusscher (TTF) model paced at a basic cycle length (BCL) of 1000 ms. The transition rates of hERG1 channel activation (α_3_), deactivation (β_3_) and recovery from inactivation (β_4_) were modified to represent a gradual increase in the relative abundance of hERG1b (see table S2 for details). B, Simulated *I*_Kr_ corresponding to the action potentials shown in A. Legend is the same as in A. C and D, Channel state occupancy during the action potential is shown for the open and inactivated states respectively. Legend is the same as in A.

As before, the hERG1 Markov model was modified to reflect the different ratios of hERG1a and hERG1b based on the factors given in table S2. Steady-state action potentials (BCL 1000 ms) are shown in figure 5A. As the relative abundance of hERG1b was increased the APD_90_ was gradually shortened from 302 ms (hERG1a) to 275 ms (hERG1b). For 20, 40, 60 and 80% hERG1b the APD_90_ was 292, 285, 285 and 279 ms respectively. In figure 5B the *I*_Kr_ corresponding to the action potentials in figure 5A are shown. The *I*_Kr_ peaked earlier and the peak amplitude became greater as the relative abundance of hERG1b was increased. Figures 5C and D show the proportion of channels in the open and inactivated states respectively during the action potential. The increase in recovery rate allowed the channels to escape the inactivated state earlier during the action potential (figure 5D). Although the maximum proportion of channels in the open state was decreased as the relative abundance of hERG1b was increased (figure 5C) the driving force on these channels were much greater due to the more depolarized membrane potential at this earlier phase resulting in the increased *I*_Kr_ amplitude. The shortening of APD in response to greater abundance of hERG1b is therefore mainly due to the increase in recovery rate.

**Figure 6:**
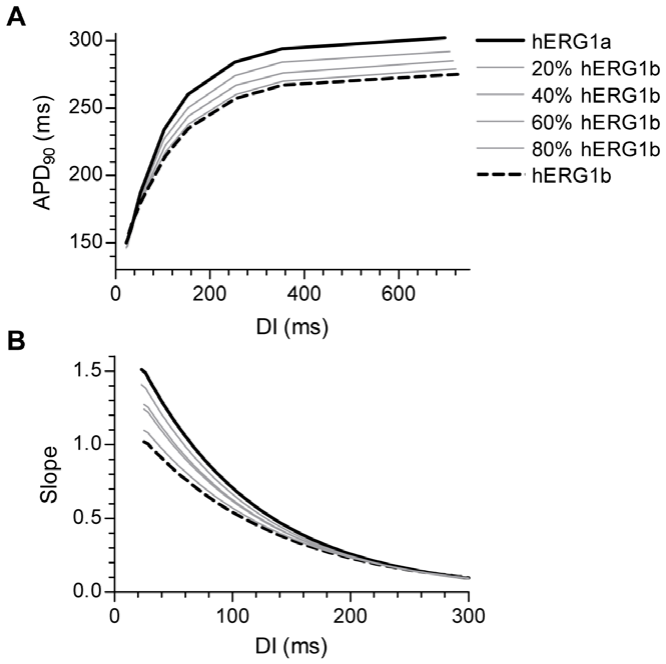
Restitution slope is modulated by the relative abundance of hERG1a and hERG1b. A, Single-cell action potential restitution. The restitution curves were obtained using an S1-S2 restitution protocol measured at a BCL of 1000 ms. The action potential duration (APD_90_) is plotted as a function of the diastolic interval (DI). The transition rates of hERG1 channel activation (α_3_), deactivation (β_3_) and recovery from inactivation (β_4_) were modified to represent a gradual increase in the relative abundance of hERG1b (see table S2 for details). B, The slopes of the restitution curves were calculated by differentiation of single-exponential functions fitted to the curves in A and plotted as a function of DI.

The TT model has been shown to reproduce data on human restitution kinetics [16]. We wanted to know whether the changes in transition rates would also affect the restitution properties. Action potential restitution was measured using a standard S1-S2 protocol. At steady-state pacing (BCL 1000 ms) an additional stimulus (S2) was introduced at successively shorter coupling intervals and the corresponding APD_90_ was determined. The relationship between APD_90_ and DI could be described by a single-exponential function. In figure 6A, the resulting restitution curves are shown. For simplicity the exponential fits are not shown. As the relative abundance of hERG1b was increased the restitution curves became gradually flatter. The slope of the restitution curve is associated with electrical stability of cardiac tissue. To study the slope of the curves in more detail, the exponential fits to the restitution curves were differentiated to obtain the slope over the whole range of DIs. The relationships between slope and DI are shown in figure 6B. The figure shows that as the relative abundance of hERG1b was increased the slope of the restitution curve was reduced markedly for DIs shorter than 200 ms. In general, the slope of the restitution curve is dependent on the balance between inward and outward currents immediately before the S2 stimulus is applied. Mechanistically, the reduced slope can therefore be explained by fewer hERG1 channels residing in the open state during late phase 3 and phase 4 of the action potential (figure 5C). Thus, the change in restitution slope is mainly due to the increase in deactivation kinetics resulting from greater hERG1b abundance.

## Discussion

A considerable variation has been observed in the ‘peak potential’ characterizing native *I*_Kr_ transients [10]. This observation may, in theory, be explained by differences in *I*_Kr_ channel kinetics. Intrinsic differences in *I*_Kr_ deactivation kinetics have been described [11] supporting the notion that considerable heterogeneity in *I*_Kr_ kinetics exists in the heart.

### Differential Expression of ERG1a and ERG1b Reproduces Properties of I_Kr_ Transients

We hypothesized that the observed variation in ‘peak potential’ of the native *I*_Kr_ transient [10] could be explained by variation in kinetic rates as a result of differential expression of ERG1 isoforms. Indeed, by varying the relative abundance of hERG1a and hERG1b we were able to manipulate the ‘peak potential’ over a wide range of potentials. We were able to do this in both mammalian cells and in *X. laevis* oocytes. In oocytes, where we could control the exact ratio of the two isoforms the ‘peak potential’ showed a very good correlation to the relative abundance of the isoforms. The differences in the absolute values of ‘peak potentials’ and peak amplitudes between ERG1 channels expressed in mammalian cells and in oocytes may be attributed to differences in the expression systems.

Compared to hERG1a channels, increased rates of both activation, deactivation and recovery from inactivation have been described for heteromeric hERG1a/b channels [8], [9]. We were able to show that the relative changes in the kinetics of deactivation and recovery from inactivation is a linear function of the relative hERG1b abundance in *X. laevis* oocytes. Interestingly, a similar relationship was not obtained for the process of activation. The reason for the non-linear relationship between activation kinetics and isoform abundance is beyond the scope of this study. However, it is worth noting that when expressed in CHO cells hERG1a/b channels display deactivation and recovery kinetics intermediate of those of hERG1a and hERG1b homomeric channels while activation kinetics of hERG1a/b are similar to hERG1b [8]. hERG1 channels are believed to be tetramers. Possibly, the number of long 1a-type N-terminals in the channel complex may play a crucial role in the activation process. Future studies focusing on molecular determinants of gating characteristics of heteromeric channels may explain these findings. Nevertheless, the systematic changes in kinetics of deactivation and recovery as a function of hERG1b abundance explain the variation in ‘peak potential’.

The absolute variation in the ‘peak potential’ observed by Gintant [10] in canine cardiomyocytes (20–30 mV) is within the range observed in our experiments (∼40 mV in HEK293 cells and ∼30 mV in *X. laevis* oocytes). Although there were differences in the experimental conditions, the data suggest a large variation in the relative abundance of ERG1a and ERG1b in native cardiomyocytes. However, our results show that the ‘peak potential’ of hERG1 channels in general occurs at more depolarized potentials as compared to the data from canine cardiomyocytes [10]. This difference can not be fully explained by differences in the temperature at which the data was obtained. It may, however, partly be attributed to species-dependent differences in *I*_Kr_ kinetics (see [18] for review). *I*_Kr_ is known to deactivate slower in canine than in human cardiomyocytes [18]. Thus, the ‘peak potential’ is expected to occur at more depolarized potentials for human ERG1 channels. Interestingly, Hua et al. [19] have shown that over-expression of hERG1a by adenoviral transfection in isolated canine cardiomyocytes shifted the ‘peak potential’ towards more depolarized potentials as compared to control. Additionally, differences in post-translational modifications of hERG1 channels and/or interactions with auxiliary proteins may play a role [18].

When we normalized the recorded currents to a value proportional to the number of expressed channels, we observed only little variation in the peak current density over the whole range of isoform ratios in both expression systems. Similarly, Gintant [10] reported only small variations in the absolute *I*_Kr_ peak density. Although speculative, taken together these data may indicate that the absolute number of ERG1 channels in the membrane of cardiomyocytes is relatively constant from cell to cell but that the *relative* abundance of ERG1a and ERG1b varies.

We have based our study on a comparison of canine *I*_Kr_ [10] and heterologously expressed hERG1a and hERG1b channels. Although our results qualitatively show that differential expression of hERG1a and hERG1b may explain the observed variation in canine *I*_Kr_, it remains to be shown to which extend post-translational modifications and/or interactions with auxiliary proteins influence the properties of native *I*_Kr_. Also, it is unknown whether a similar variation is present in the human heart. Given the relatively high abundance of hERG1b in the human heart [5], [8] it seems plausible to suggest that at least some degree of variation is present. Interestingly, the existence of separate promoter regions for hERG1a and hERG1b has been described [20] providing a potential mechanism for such differences. Therefore, differences in the relative abundance of hERG1a and hERG1b may underlie regional heterogeneity of *I*_Kr_ kinetics. Future studies looking into these issues are needed to fully establish the importance of ERG1b in the human heart. To this end, our results suggest that it is the relative (to ERG1a) rather than the absolute abundance of ERG1b that determines the properties of *I*_Kr_.

### Functional Implications of ERG1b in the Heart

To address the possible functional implications of differential expression of hERG1a and hERG1b in the heart we conducted a series of computer simulations. First, we used a previously published hERG1 Markov model [15] to reproduce the experimental data. In our simulations we changed the kinetic rates of the Markov model by factors corresponding to the relative changes in kinetics observed in the *in vitro* experiments. Qualitatively, the model reproduced the experimental data well, especially considering that the intrinsic kinetics of the model were somewhat different than the experimental observations (single vs. double-exponential kinetics of deactivation). Second, the Markov model was introduced into the TT model of the ventricular cardiomyocyte. The results of the ventricular cardiomyocyte simulations showed that kinetic changes in *I*_Kr_ corresponding to increases in the relative abundance of hERG1b resulted not only in gradually shorter steady-state APD but also in flatter restitution curves. Mechanistically, inspection of the changes in channel state occupancy revealed that the shortening of APD was mainly due to the increase in recovery rate. Similarly, the decrease in restitution slope was mainly a result of the increase in deactivation rate.

The existence of a transmural APD gradient has been well established and has been attributed to differences in *I*_K_ density [21]–[23]. Our results show that differences in *I*_Kr_ kinetics, especially differences in kinetics of recovery from inactivation, may also contribute to APD dispersion. It is therefore interesting to note that in the study by Szabo et al. [11] the shortest APDs were recorded in the epicardial cells where also the deactivation kinetics of *I*_Kr_ were fastest. Assuming that faster deactivation rate is due to greater relative abundance of ERG1b, *I*_Kr_ in these cells would be expected also to display a faster rate of recovery from inactivation. In our simulations such differences are predicted to shorten APD, suggesting that differences in *I*_Kr_ kinetics may indeed contribute to the APD dispersion observed *in vivo*.

APD restitution describes the relationship between APD and DI. When the slope of the restitution curve is steep, APD is very sensitive to even small changes in DI and as a result APD alternans may develop. Accordingly, steep restitution slopes has been associated with an increased risk of ventricular fibrillation [24]–[26]. It has also been shown that the restitution properties vary between different regions of the heart [27]–[29]. Although restitution heterogeneity has been described in the normal heart [27], increased heterogeneity may provide a substrate for arrhythmia in diseased hearts [29]. The mechanism of APD restitution is complex and likely involves several factors. Previously, the relative densities of *I*_Kr_ and *I*_Ks_ have been implicated in determining restitution slope [30], [31]. Also, calcium dynamics are involved as blockade of L-type calcium channels flatten restitution [26]. Our results suggest that also differences in the intrinsic kinetics of *I*_Kr_ resulting from variation in the relative abundance of hERG1a and hERG1b may play a role.

In summary, the results suggest that by modulating the relative expression of ERG1a and ERG1b, a given cell can control the kinetics of *I*_Kr_ resulting in modulation of both APD and the slope of the restitution curve. The results show that cells with a low relative abundance of ERG1b will have relatively long baseline APD and steep restitution slopes. Cells with a high relative abundance of ERG1b will have shorter baseline APD and less steep restitution slopes. Interestingly, a similar relationship between baseline APDs and restitution kinetics have been observed in native cardiac cells [27], [32]. Thus, the predicted effect of regional variation in the relative abundance of ERG1a and ERG1b agree well with existing findings in cardiac tissue. As such, regional differences in the relative abundance of ERG1a and ERG1b may represent a potential mechanism underlying the heterogeneity observed in both APD and APD restitution kinetics. This is, to our knowledge, the first time that intrinsic variation in *I*_Kr_ kinetics has been suggested to play a role in heterogeneity of APD restitution. However, as this association is based on indirect evidence from heterologous expression and computational modeling experiments, more studies examining the relationship between the relative abundance of ERG1 isoforms and restitution kinetics in cardiac tissue are needed to determine if this predicted association is also evident *in vivo*.

In conclusion, our results show that differential expression of hERG1a and hERG1b can reproduce the observed variation in the characteristics of native *I*_Kr_. Thus, based on a functional comparison between heterologously expressed hERG1 channels and native *I*_Kr_, the results demonstrate that variations in the relative abundance of ERG1 isoforms are likely present in the heart. Computer simulations suggest that differences in the relative abundance of hERG1a and hERG1b translate into differences in APD and APD restitution on the cellular level. Consequently, we propose that differences in the relative abundance of hERG1a and hERG1b may represent a potential mechanism underlying regional heterogeneity of both APD and APD restitution observed in mammalian hearts. Our results add to the growing body of evidence that ERG1b contributes significantly to the generation of cardiac *I*_Kr_ and plays an important role in cardiac electrophysiology.

## Supporting Information

Table S1 (0.10 MB PDF)

Table S2 (0.08 MB PDF)

Figure S1 Correlation between macroscopic channel kinetics and isoform abundance. The relative change in the kinetic parameters of deactivation (A, fast component; B, slow component), recovery from inactivation (C) and activation (D) as compared to hERG1a (0% hERG1b) was calculated based on the values in table S1 and plotted as a function of hERG1b cRNA abundance. The solid lines indicate the linear correlations. The correlation coefficients are also shown. In C, the dotted line and the open square indicate the extrapolated value for hERG1b.
(0.26 MB TIF)

Figure S2 Comparison of deactivation kinetics for observed and theoretical currents. A-D, Calculations of observed and theoretical time course of current decay are shown for different relative abundances of hERG1b as indicated. The observed current decay (Exp, solid lines) in each situation was calculated from the observed macroscopic deactivation properties (table S1). The theoretical current decay (Theory, dotted lines) was calculated under the assumption that only hERG1a and hERG1b channels were formed. Notice that for all comparisons the observed currents decay faster than the theoretical predictions.
(0.30 MB TIF)
