# Supplementary material for: Differential Expression of hERG1 Channel Isoforms Reproduces Properties of Native I Kr and Modulates Cardiac Action Potential Characteristics
Source: PLoS One. 2010 Feb 2;5(2):e9021. doi: 10.1371/journal.pone.0009021 (PMC2814852; doi:10.1371/journal.pone.0009021)
Supplement: Table S1 — (0.10 MB PDF) [file pone.0009021.s001.pdf]

Table S1. Time constants of hERG1 channel kinetics measured in *X. laevis* oocytes

	Activation	Deactivation			Recovery
	τ_{act} (ms)	τ_{fast} (ms)	τ_{slow} (ms)	$A_{fast}/(A_{fast}+A_{slow})$	τ_{rec} (ms)
hERG1a	190.4 ± 9.3	443.6 ± 17.9	2272.5 ± 69.7	0.27 ± 0.01	9.4 ± 0.2
20% hERG1b	129.9 ± 5.4	117.2 ± 4.7	801.4 ± 24.9	0.61 ± 0.01	7.4 ± 0.3
40% hERG1b	95.2 ± 5.3	80.8 ± 3.3	567.3 ± 21.7	0.73 ± 0.01	6.0 ± 0.2
60% hERG1b	129.7 ± 8.9	69.1 ± 2.1	458.0 ± 9.9	0.79 ± 0.01	6.0 ± 0.2
80% hERG1b	122.8 ± 7.0	47.3 ± 1.7	310.1 ± 15.0	0.85 ± 0.01	5.0 ± 0.1
hERG1b	122.4 ± 10.7	31.8 ± 0.9	272.5 ± 9.6	0.92 ± 0.00	na.
Relative change in τ (compared to hERG1a)					
hERG1a	1	1	1		1
20% hERG1b	1.5	3.8	2.8		1.3
40% hERG1b	2.0	5.5	4.0		1.6
60% hERG1b	1.5	6.4	5.0		1.6
80% hERG1b	1.5	9.4	7.3		1.9
hERG1b	1.6	14.0	8.3		2.1 *

All data are shown as mean ± SEM. Kinetics of activation were determined at 0 mV. Kinetics of deactivation and recovery from inactivation were determined at -60 mV.

* This value is an estimate based on extrapolation from the linear correlation; r^2=0.94.
