# Supplementary material for: Differential Expression of hERG1 Channel Isoforms Reproduces Properties of Native I Kr and Modulates Cardiac Action Potential Characteristics
Source: PLoS One. 2010 Feb 2;5(2):e9021. doi: 10.1371/journal.pone.0009021 (PMC2814852; doi:10.1371/journal.pone.0009021)
Supplement: Table S2 — (0.08 MB PDF) [file pone.0009021.s002.pdf]

Table S2. Changes to model transition rates

	α_3	β_3	β_4
hERG1a	1	1.0	1.0
20% hERG1b	1.5	3.8	1.3
40% hERG1b	1.5	5.5	1.6
60% hERG1b	1.5	6.4	1.6
80% hERG1b	1.5	9.4	1.9
hERG1b	1.5	14.0	2.1

The numbers are the corresponding factorial changes to the transition rates in the hERG1 Markov model.
