# Supplementary figures and images for: Differential Expression of hERG1 Channel Isoforms Reproduces Properties of Native I Kr and Modulates Cardiac Action Potential Characteristics
Source: PLoS One. 2010 Feb 2;5(2):e9021. doi: 10.1371/journal.pone.0009021 (PMC2814852; doi:10.1371/journal.pone.0009021)

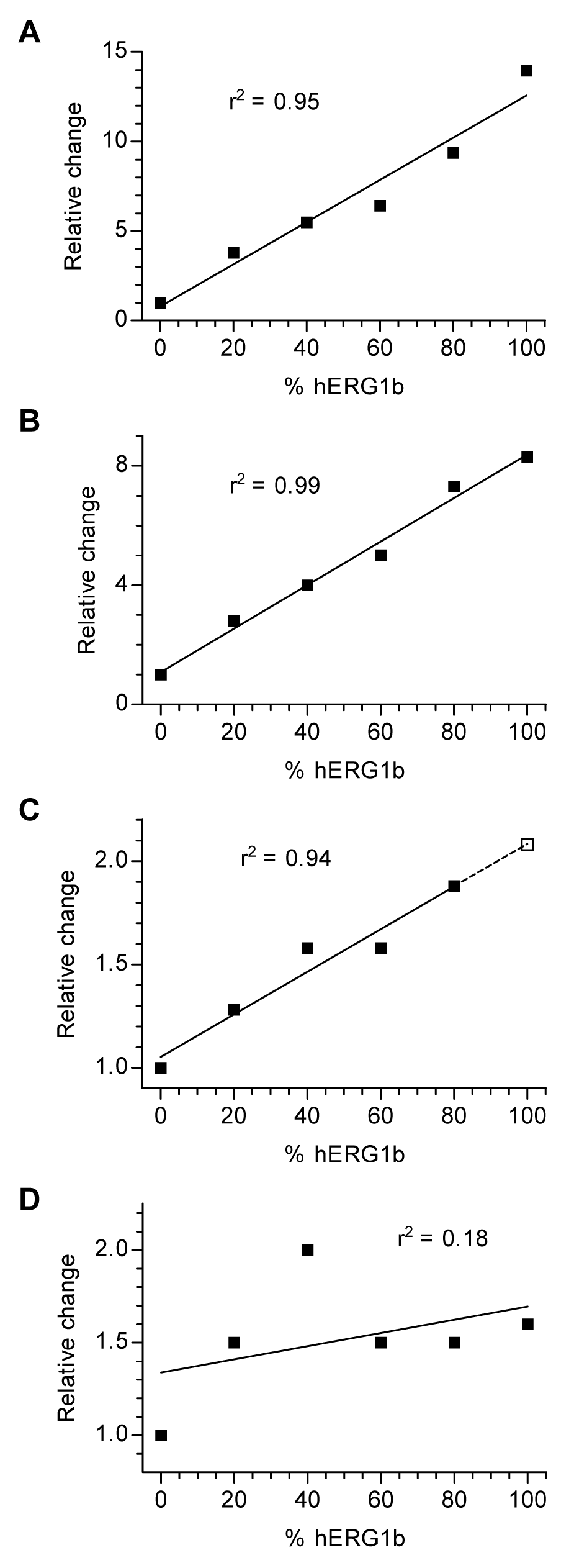

Supplement: Figure S1 — Correlation between macroscopic channel kinetics and isoform abundance. The relative change in the kinetic parameters of deactivation (A, fast component; B, slow component), recovery from inactivation (C) and activation (D) as compared to hERG1a (0% hERG1b) was calculated based on the values in table S1 and plotted as a function of hERG1b cRNA abundance. The solid lines indicate the linear correlations. The correlation coefficients are also shown. In C, the dotted line and the open square indicate the extrapolated value for hERG1b. (0.26 MB TIF) [file pone.0009021.s003.tif]

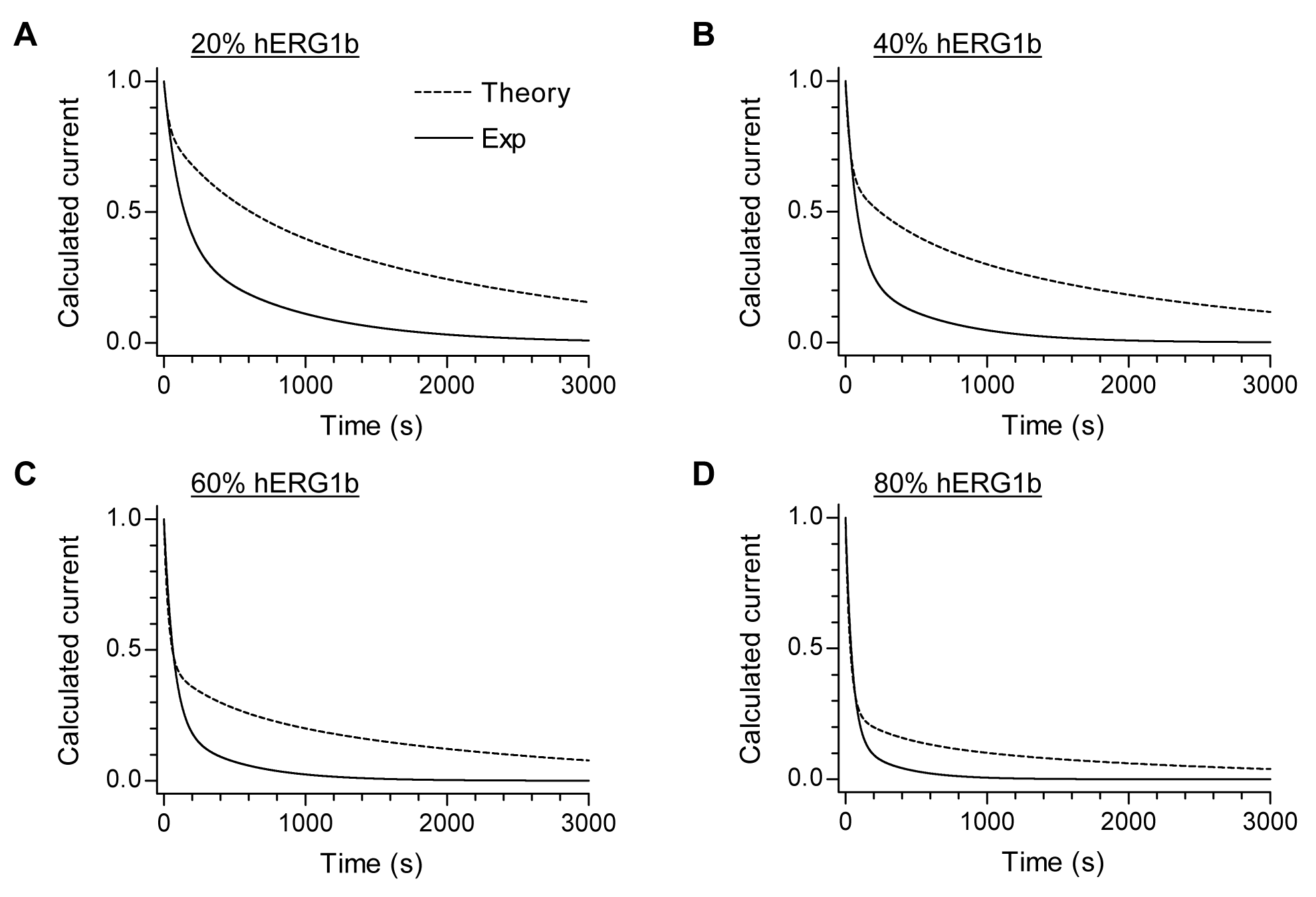

Supplement: Figure S2 — Comparison of deactivation kinetics for observed and theoretical currents. A-D, Calculations of observed and theoretical time course of current decay are shown for different relative abundances of hERG1b as indicated. The observed current decay (Exp, solid lines) in each situation was calculated from the observed macroscopic deactivation properties (table S1). The theoretical current decay (Theory, dotted lines) was calculated under the assumption that only hERG1a and hERG1b channels were formed. Notice that for all comparisons the observed currents decay faster than the theoretical predictions. (0.30 MB TIF) [file pone.0009021.s004.tif]
